# Telemedicine and other care models in pediatric rheumatology: an exploratory study of parents’ perceptions of barriers to care and care preferences

**DOI:** 10.1186/s12969-017-0184-y

**Published:** 2017-07-11

**Authors:** Danielle R. Bullock, Richard K. Vehe, Lei Zhang, Colleen K. Correll

**Affiliations:** 1 Division of Pediatric Rheumatology, Department of Pediatrics, University of Minnesota, East Bldg Rm M668, 2450 Riverside Ave, Minneapolis, MN 55454 USA; 2 Clinical and Translational Sciences Institute, University of Minnesota, 717 Delaware Street SE, Minneapolis, MN 55414 USA

**Keywords:** Access, Barriers, Outreach, Pediatric rheumatology, Telemedicine

## Abstract

**Background:**

The United States pediatric rheumatology workforce is committed to a mission of providing children access to pediatric rheumatology care. With a limited number and distribution of pediatric rheumatologists, telemedicine has been proposed as one way to meet this mission, yet the adoption of this modality has been slower than expected. The purpose of this study was to explore the parent perspective on barriers to accessing pediatric rheumatology care and to explore the acceptability of telemedicine and other alternative care models.

**Methods:**

Over a period of six weeks, all new and return English-speaking parents/guardians of patients visiting a single center were offered an opportunity to complete a survey which assessed barriers to care and interest in alternative models of care. Responses were analyzed using descriptive statistics.

**Results:**

Survey response rate was 72% (159/221). Twenty-eight percent (45/159) traveled more than three hours to the pediatric rheumatology clinic, and 43% (65/152) reported travel as inconvenient. An overwhelming majority of respondents (95%, 144/152) reported a preference for in-person visits over the option of telemedicine. This preference was similar regardless of whether respondents reported travel to the clinic as inconvenient vs convenient (inconvenient 92%, 60/65; convenient 97%, 84/87; *p* = 0.2881) and despite those reporting travel as inconvenient also reporting greater difficulty with several barriers to care. Those familiar with telemedicine were more likely to report a preference for telemedicine over in-person visits (27%, 3/11 vs 3%, 4/140; *p* = 0.0087). The option of an outreach clinic was acceptable to a majority (63%, 97/154); however, adult rheumatology and shared-care options were less acceptable (22%, 35/156 and 34%, 53/156 respectively).

**Conclusion:**

Among survey respondents, in-person visits were preferred over the option of telemedicine, even when travel was noted to be inconvenient. Telemedicine familiarity increased its acceptability. Outreach clinics were acceptable to a majority. Ultimately, the parent perspective can shape acceptable ways to address barriers and provide accessible care.

**Electronic supplementary material:**

The online version of this article (doi:10.1186/s12969-017-0184-y) contains supplementary material, which is available to authorized users.

## Background

The pediatric rheumatology (PR) workforce is committed to a mission of providing children with access to care and superior clinical outcomes [1]. With rheumatic diseases affecting more than 300,000 children in the United States (US), the PR workforce of approximately 300 pediatric rheumatologists, primarily concentrated at academic institutions, faces many challenges in achieving this mission [2]. Recognizing a clear deficit in the number and distribution of pediatric rheumatologists, a 2007 report to Congress recommended several remedies, including piloting telemedicine networks [2]. This was echoed in a more recent 2011 examination of the PR workforce [1], and telemedicine has also been viewed as a promising solution to access concerns in adult rheumatology [3]. Other solutions, such as shared-care, in which patients are co-managed with community-based physicians, have also been proposed [2].

Despite probable benefits, telemedicine adoption has been slower than expected. Studies in other areas of medicine, including but not limited to adult rheumatology and other pediatric subspecialties, have reported high levels of satisfaction with telemedicine [3–5]; however, the acceptability is not universal. A recent survey which sampled households in rural Montana reported that a high percentage of respondents were unequivocally averse to using telemedicine for their healthcare, despite the inconvenience of in-person visits [6]. Additional concerns include the clinical effectiveness of telemedicine in a specialty highly dependent on the physical examination, cost effectiveness, and the quality of the patient-physician relationship, all of which would need to be addressed for successful adoption in the PR community [7–9].

Few PR clinics currently use telemedicine, and there is only one publication describing its use for PR patients [10]. This study found that telemedicine interest was greater for those living further from the tertiary care center and among those spending more time away from work [10]. As additional PR clinics consider telemedicine adoption, more research is needed to understand how certain factors might contribute to or hinder telemedicine success. In other areas of medicine, various predictors of and barriers to telemedicine adoption have been explored (e.g. age, Internet use, digital literacy, rural residence, perceived privacy), yet conclusive evidence on many of these remains elusive [11, 12]. Additionally, because telemedicine is unlikely to be appropriate for all patients, the acceptability of other care models should also be explored.

The purpose of this survey study was to delineate barriers to PR care and to explore the acceptability of alternative PR care models, with an emphasis on telemedicine, among a population of PR patients in the Upper Midwest. Specific objectives were 1) to explore the parent/guardian perspective on barriers to accessing PR care and to identify factors associated with discrepant barrier perceptions, 2) to assess whether a telemedicine option, if available, might be preferred over in-person care, 3) to identify factors influencing opinions about telemedicine use, and 4) to examine the acceptability of other care models, including outreach clinics, adult rheumatology care, and shared-care.

## Methods

### Patients

All new or return English-speaking parents/guardians of patients who visited the University of Minnesota PR Clinic during a six-week period in 2015 were eligible. This was a convenience sample since only those coming to the clinic during this time period were offered the survey. Patients 18 years of age or older were allowed to complete the survey independently.

### Survey design and administration

Review of the PR workforce literature, barriers to accessing care, and telemedicine informed development of the survey, which was deemed exempt by the University of Minnesota Institutional Review Board (IRB). Parents/guardians were given a paper copy of the survey at the time of their child’s PR appointment. Survey responses included multiple choice answers as well as “choose all that apply” options. A five dollar gift card was given for survey completion.

Survey questions included demographics as well as the child’s diagnosis, length of time the child was a patient in the clinic, and travel time to the clinic. A subjective assessment of whether travel was convenient vs inconvenient was also included. Eleven potential barriers to accessing care were assessed: availability of appointment dates/times, travel time/distance, need for lodging, adequate transportation, driving in the metropolitan, direct costs (medical bills, medications, etc.), indirect costs (gas, hotel, food, etc.), child missing school, parent/guardian missing work, arranging care for other children, and insurance approval for visits. These were scored on a 0 (no difficulty) to 10 (difficult enough to stop you from getting health care for your child) scale. Visit preference (in-person vs telemedicine), familiarity with telemedicine (personal telemedicine use and/or having a close family member or friend who had used telemedicine), and subjective assessment of telemedicine quality were examined. Finally, the acceptability of outreach clinics, adult rheumatology, and shared-care were explored. Since the care model for the University of Minnesota PR Division at the time of this survey consisted of only in-person visits at the academic center, the respondent assessment of alternative care models was based on a brief description of these modalities and any personal experience outside of the clinic. A copy of the survey can be viewed as an additional file (see Additional file 1).

### Survey analysis

Responses were summarized using descriptive statistics, expressed as frequencies and percent, or mean ± standard deviation (SD)/median and interquartile range (IQR). Due to small, non-normally distributed data or small sample size, Kruskal-Wallis test was used for group comparisons of continuous variables. For categorical variables, Fisher’s exact test was used to compare groups. All analyses were done using the SAS system (v. 9.3; SAS Institute, Cary, NC). *P*-values were two-sided with <0.05 considered statistically significant. Bonferroni adjustment was applied for multiple comparisons. Missing data were excluded from analyses using pairwise deletion, and the values in the reported results reflect that some respondents did not answer all survey questions.

## Results

### Patient characteristics

The response rate was 72% (159/221). Twenty-four percent (38/159) of the respondents were parents/guardians of new patients to the clinic; the remainder were follow-up visits. Patient characteristics are listed in Table 1. The majority were parents/guardians of adolescent Caucasian patients and had private insurance. Most patients carried a diagnosis of juvenile idiopathic arthritis (JIA). Geographic distribution of patients included Minnesota, Wisconsin, North Dakota, and South Dakota with 52% (82/159) traveling more than one hour and 28% (45/159) traveling more than three hours to the clinic. Due to survey anonymity, characteristics of non-responders could not be assessed.

**Table 1:** Patient characteristics

Characteristic	Classification	N (%)^a^
Age	< 6	15 (9.4)
	6–12	65 (40.9)
	≥ 13	76 (47.8)
Ethnicity	White	136 (85.5)
	Non-white	21 (13.2)
Insurance	Private	113 (71.1)
	Public	43 (27.0)
	None	1 (0.6)
Primary diagnosis	Juvenile idiopathic arthritis	90 (56.6)
	Not yet determined	26 (16.4)
	Other	10 (6.3)
	Juvenile dermatomyositis	7 (4.4)
	Uveitis/iritis	6 (3.8)
	Chronic recurrent multifocal osteomyelitis	4 (2.5)
	Spondyloarthropathy	3 (1.9)
	Systemic lupus erythematosus	3 (1.9)
	Mixed connective tissue disease	3 (1.9)
	Periodic fever	2 (1.3)
	Psoriasis	1 (0.6)
	Reactive arthritis	1 (0.6)
	Behcet’s disease	1 (0.6)
	Amplified musculoskeletal pain	1 (0.6)
Travel time	≤ 1 h	76 (47.8)
	> 1 h - ≤ 3 h	37 (23.3)
	> 3 h - ≤ 6 h	37 (23.3)
	> 6 h	8 (5.0)

^a^Percentages are out of 159 survey respondents. Some respondents did not answer all questions

### Barriers to accessing care

The highest scoring barrier (0 being none and 10 being great) was the child missing school with a median of 2.50 (IQR 0.50–5.00), followed by travel time/distance and parent/guardian missing work, each with a median of 2.00 (IQR 0.50–5.00 and 0.00–5.00, respectively). Other median scores were as follows: availability of appointment dates/times (median 0.50, IQR 0.00–2.00), need for lodging (0.00, 0.00–1.00), adequate transportation (0.00, 0.00–0.50), driving in the metropolitan (0.50, 0.00–3.00), direct costs (0.50, 0.00–3.00), indirect costs (0.50, 0.00–3.00), arranging care for other children (0.00, 0.00–1.50), insurance approval for visits (0.00, 0.00–1.00).

Pairwise comparison revealed a statistically significant difference in arranging care for other children, with families whose child was less than six years old reporting a higher score (median 1.50, IQR 0.00–6.00) compared to families whose child was 13 years or older (0.00, 0.00–0.50; *p* = 0.016). Those with non-private insurance scored travel time/distance as a greater barrier than those with private insurance (median 3.75, IQR 0.75–5.50 vs 2.00, 0.50–5.00; *p* = 0.0398). Other pairwise comparisons between barrier scoring and patient factors (age, insurance, ethnicity, and diagnosis) revealed no significant differences.

Those who indicated travel as inconvenient vs convenient reported significantly greater difficulty with several barriers: travel time/distance (median 5.00, IQR 2.50–6.00 vs 1.00, 0.00–2.50; *p* < 0.0001), need for lodging/housing (0.25, 0.00–4.00 vs 0.00, 0.00–0.50; *p* = 0.0047), driving in the metropolitan (2.75, 0.00–5.00 vs 0.00, 0.00–1.00; *p* < 0.0001), indirect costs (2.50, 0.00–5.00 vs 0.00, 0.00–1.00; *p* < 0.0001), parent missing work (3.00, 1.00–6.00 vs 1.00, 0.00–3.00; *p* < 0.0001), child missing school (4.00, 1.25–6.25 vs 2.00, 0.50–3.50; *p* = 0.0008), and arranging care for other children (0.25, 0.00–5.00 vs 0.00, 0.00–0.50; *p* = 0.0219). The overall distribution of travel times was different depending on whether care was perceived as inconvenient vs convenient (*p* < 0.0001). For those reporting travel as convenient, 71% traveled an hour or less, 17% traveled more than an hour to 3 h or less, 10% traveled more than 3 h to 6 h or less, and 1% traveled more than 6 h. For those reporting travel as inconvenient, 18% traveled an hour or less, 34% more than an hour to 3 h or less, 37% more than 3 h to 6 h or less, and 11% more than 6 h.

### Telemedicine

**Fig. 1:**
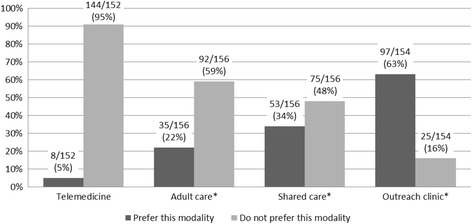
Acceptability of alternative care models. Legend: * Respondents with neutral interest in these alternative care models are not represented in this figure

An overwhelming majority of respondents (95%, 144/152) reported a preference for in-person visits over the option of telemedicine (Fig. 1). This preference was similar regardless of whether travel was inconvenient (inconvenient 92%, 60/65 vs convenient 97%, 84/87; *p* = 0.2881) and despite the inconvenient group reporting greater difficulty with several barriers.

**Fig. 2:**
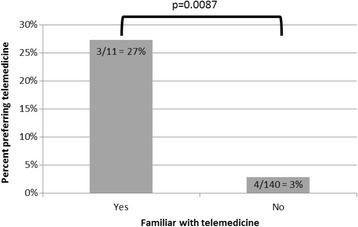
Respondents familiar with telemedicine more accepting of this modality

Only 8% (13/158) of respondents reported familiarity with telemedicine. The majority of respondents (75%, 115/154) felt that they had inadequate knowledge about telemedicine to compare its quality to in-person visits; however, those familiar with telemedicine were more likely to report it as equal to or better than in-person visits (42%, 5/12 vs 8%, 11/142; *p* = 0.0033) and were also more likely to report a preference for telemedicine over in-person visits (Fig. 2).

The small number of respondents preferring a telemedicine option scored travel time/distance as a greater barrier compared to those preferring in-person visits (median 5.00, IQR 4.00–6.25 vs 2.00, 0.50–5.00; *p* = 0.0142). No other differences in barrier scores were detected between those preferring the option of telemedicine visits over in-person visits.

Neither views on telemedicine quality compared to in-person visits nor preference for in-person visits significantly differed by patient demographics, insurance type, length of time the child was a patient in the clinic, or travel time. Additionally, there was no detectable difference in median days of internet or video chat use between groups.

### Other care models

Interest in adult rheumatology care (22%, 35/156) and shared-care management with an adult rheumatologist or primary physician (34%, 53/156) was low (Fig. 1). In contrast, 63% (97/154) indicated that outreach clinics would be an acceptable alternative to current tertiary center care (Fig. 1). There was no detectable difference in willingness to participate in a shared-care model of care based on the perceived comprehensiveness of the child’s primary care clinic.

Travel time/distance was consistently scored as a significantly greater barrier among those interested vs not interested in alternative care models – adult rheumatology care (median 5.00, IQR 2.00–6.00 vs 1.75, 0.00–5.00; *p* = 0.0054), shared-care (4.50, 1.00–5.00 vs 1.00, 0.00–5.00; *p* = 0.0031), and outreach clinics (4.25, 1.00–5.50 vs 0.00, 0.00–1.00; *p* < 0.0001). Direct costs, indirect costs, driving in the metropolitan, parent missing work, and child missing school were also scored as greater barriers among those interested in certain alternative care models (Additional file 2).

Separate from the travel time/distance barrier assessment, self-reported total travel time was also examined. Interest in adult rheumatology care did not differ based on travel time. For shared care and outreach clinics, the distribution of self-reported travel times differed depending on interest in these care models (Table 2).

**Table 2:** Interest in shared care and outreach according to travel time

		Travel Time				
Care Model	Agree or Disagree^a^	≤ 1 h N (%)	> 1 - ≤ 3 h N (%)	> 3 - ≤ 6 h N (%)	> 6 h N (%)	*P*-value
Shared Care	Agree	17 (32)	17 (32)	16 (30)	3 (6)	0.0387
	Disagree	43 (58)	12 (16)	14 (19)	5 (7)	
Outreach	Agree	38 (40)	23 (24)	31 (32)	4 (4)	0.0041
	Disagree	16 (64)	7 (28)	0 (0)	2 (8)	

^a^Those with neutral interest are not displayed in this table

## Discussion

Health care access is a complex issue with many contributing factors. Availability and accessibility are just part of a bigger picture [13], yet clearly affect PR referral decisions among primary pediatricians, as shown in two recent survey studies [14, 15]. While solutions have been recommended to help remedy these concerns [1, 2], including piloting telemedicine networks and enhancing the rheumatology training of adult rheumatologists and primary care providers, no previous studies have examined the parent perspective on barriers to PR care or the perceived acceptability of proposed solutions.

We found that respondents in this survey reported few barriers to PR care. Certain features, such as having younger children or viewing travel as inconvenient, may affect barrier perceptions and should certainly be taken into account when considering a family’s needs. This does not, however, necessarily translate into acceptance of alternative models. While some barriers, such as travel time/distance, were scored higher among those interested in alternative models, a majority of respondents still preferred the current care model. Even when travel was inconvenient, respondents strongly preferred in-person visits over the idea of telemedicine, and this was true even though several barriers were scored higher by those reporting travel as inconvenient. With limited resources, we must question whether improving care for children with rheumatic diseases should focus on expanding the reach of care or enriching current care models. It remains to be seen what barriers would truly be lessened by different alternative care models. As models are implemented, further research is needed to better quantify the trade-offs. A recent PR telemedicine study, for example, showed patients and families using telemedicine missed less time from school or work compared to usual tertiary center care [10]. Understanding, quantifying, and transparently sharing the ways in which alternative care models may reduce such barriers will be vital to gaining acceptability.

While most families scored barriers to care relatively low and reported a preference for the current care model, it is imperative to recognize that these preferences do not apply to all families. We should not underestimate the role that accessibility might play for some families. Though not a majority, accessibility was still a notable barrier among our survey respondents and specifically addressing this concern could contribute to the success of alternative models for certain families. Travel time/distance – an accessibility issue – was reported as a greater barrier among those who expressed interest in telemedicine, adult rheumatology care, shared-care, and outreach clinics. Further work is needed to understand what contributes to these barrier perceptions and when such perceptions are enough to tip the balance toward acceptance of alternative care models. Furthermore, it is easy to assume that an objective measure of access, such as distance from a care center, determines barriers and informs potential solutions. Our results demonstrate that such an approach fails to account for differences between parent perspective and objective measures. As mentioned, the acceptability of all alternative care models varied based on *parent-reported* barriers in travel time/distance. Actual travel times, however, gave more varied results. While there were differences in the actual travel time distribution for shared care and outreach clinics, we were unable to identify variations in acceptability of telemedicine and adult rheumatology care based on travel time. Additionally, while identifying travel time/distance as a barrier seemed to be one factor contributing to perceptions of whether travel was convenient vs inconvenient, other barriers also seemed to contribute to this perception. Parent perceptions about care accessibility and what contributes to this, then, are equally as important as the objective measures we often use as a proxy, and we need to be cautious about how we apply such proxies. For example, providers sometimes refer patients to specialties more geographically accessible than PR due to the provider-perceived barrier of a long travel distance [14]. It is not clear that all parents would perceive this barrier in the same way, and we must consider the shortcoming of making such assumptions and find more effective ways to include family perspective in care decisions.

The assessment of additional alternative care models revealed that a majority of respondents were agreeable to outreach clinics. Adult rheumatology and shared-care, on the other hand, were not preferable by the majority, but some respondents were still agreeable to these. Our survey also revealed low preference for telemedicine overall; however, familiarity positively influenced both a preference for a telemedicine option and the assessment of telemedicine quality. We were unable to identify other potential predictors of telemedicine success. These findings, together with the fact that most respondents felt that they had inadequate knowledge about telemedicine to comment on its quality, highlights the vital need to focus on buy-in from those who might use this technology. Low preference does not exclude telemedicine use but informs us that acceptance may not be as quick or easy as anticipated. In thinking about telemedicine adoption, then, it is imperative to tailor implementation accordingly. A rapid expansion without the necessary buy-in and exposure may result in a telemedicine failure. In contrast to other predictors of and barriers to telemedicine adoption [10–12], telemedicine familiarity is modifiable and should be a focus of future access efforts. Studies suggest that among those actually using the technology, acceptance is high [3, 4, 10], and hesitation is a characteristic of those not necessarily familiar with telemedicine [6]. The initial barrier seems to be convincing those who are unfamiliar that telemedicine is an acceptable and effective alternative. High quality research in this area is lacking [7, 9], and as we strive to increase telemedicine familiarity, we have an obligation to ensure that convenience does take precedence over quality. It will also be critical for the PR community to consider how additional technologies, such as platforms which capture patient-reported outcomes and wearable devices, might be integrated into patient care.

An important limitation of our survey is sample bias since only those who came to our PR clinic were surveyed. These families may have different views on barriers to care and acceptable alternatives than those who never make it to our clinic. Potentially, those who could be helped most by alternative care models were not captured in this study because they never made it to the PR clinic. There is unfortunately no way to accurately identify such patients. While this sample bias is an important consideration when it comes to the generalizability of results, the intent of this survey was to capture the views of a population of already established PR patients in the Upper Midwest and to demonstrate how such views might be used, in conjunction with the literature and other objective measures, to inform thinking about care expansion. It is possible that the preference for current care at our center could be due to our sampling and/or inexperience with the other care modalities. Survey results were not intended to preclude use of other care modalities but to highlight the importance of knowing where current preferences lie, to understand where there may be resistance to care expansion, and to appreciate factors which might make expansion of alternative care models more acceptable. It is important to note that the entirety of factors which contribute to parent perceptions of care cannot be captured in a single survey. To more completely understand parent perceptions, it will be helpful in future studies to also include qualitative methods. Additionally, given limited experience with telemedicine among respondents in our survey, it will be useful to also assess telemedicine perceptions at a clinic where patients are more familiar with this modality. This, too, could include qualitative methods to identify themes around telemedicine acceptance and/or hesitation.

We had a relatively homogenous group of patients that may not be representative of other PR centers. Centers with demographics differing greatly from ours, such as centers caring primarily for patients with public insurance, could find differing preferences. The wide catchment area and long travel times for many patients coming to our clinic makes exploration of alternative care models particularly relevant for our center. This may not be the case for other centers, although it is interesting that our survey identified parent-reported travel barriers and travel inconvenience as more meaningful than actual travel time. Thus, barrier perceptions and acceptability of alternative care models can be a useful assessment at any center and can help shape expansion of care. PR centers might also consider what modalities best fit with their intended mission. Telemedicine, for example, might be implemented in parts of the world where there are no pediatric rheumatologists, and some PR centers may have interest in developing such partnerships.

Finally, small numbers, such as the small number of respondents preferring telemedicine, made it difficult to detect differences between groups in our survey. Certain distributions were weighted unevenly, such as a large percentage of patients traveling less than one hour and only a few traveling more than six hours, thus skewing median barrier scores.

## Conclusions

Effectively deploying a limited workforce is an enduring issue and requires appropriately guided stewardship of valuable resources. As we seek to remedy the PR access concerns, we must assess the readiness to engage the solutions. Given a strong preference for current care at our center, it is important that any alternative models compliment rather than detract from current offerings. Of several alternatives offered to bridge pediatric rheumatology access gaps, outreach clinics were the most preferred option at our center. While not a majority, a portion of respondents also expressed interest in adult rheumatology care and shared care. Moving forward, it will be important to gain a better understanding of what factors, in addition to the barriers to care assessed here, might cause patients to favor certain modalities over others. Telemedicine was a less favored option which, again, would not exclude its use, but further work should be done to understand which types of patients are the best fit for this modality and how exposure to this technology might change perceptions over time. It will also be important to explore the possibility of combined models (e.g. alternating current care and telemedicine visits) and assess what visit types (e.g. new encounter vs return) or diagnoses might be more amendable to alternative care models. As our center plans and pilots both outreach clinics and telemedicine, we are reminded that our mission is multifaceted – providing children with access to care as well as ensuring superior clinical outcomes [1]. In accomplishing this mission, it will be important to measure alternative models against current care, both in terms of family perceptions and objective clinical measures.

## Additional files

Additional file 1: Pediatric Rheumatology Survey. This is a copy of the survey which was completed by parents. (DOCX 177 kb)

Additional file 2: Acceptability of alternative care models shows variability according to barrier perceptions. This is supplementary table which shows in more details how different barriers to care were scored among those who were/were not interested in alternative care models. (DOCX 17 kb)

## Abbreviations

IQR: Interquartile range
IRB: Institutional review board
JIA: Juvenile idiopathic arthritis
PR: Pediatric rheumatology
SD: Standard deviation
US: United States
