# Supplementary material for: Telemedicine and other care models in pediatric rheumatology: an exploratory study of parents’ perceptions of barriers to care and care preferences
Source: Pediatr Rheumatol Online J. 2017 Jul 11;15:55. doi: 10.1186/s12969-017-0184-y (PMC5504634; doi:10.1186/s12969-017-0184-y)
Supplement: Supplementary file 1 — Pediatric Rheumatology Survey. This is a copy of the survey which was completed by parents. (DOCX 177 kb) [file 12969_2017_184_MOESM1_ESM.docx]

Thank you for taking this needs assessment survey.  Please answer the questions to the best of your ability. If this is your child’s first visit in the Pediatric Rheumatology clinic, you may not be familiar with some of the resources that we offer. If you are unable to answer a specific question, you may choose an unknown/undecided option or skip the question.

| **1.** | **Approximately how long has your child been seen by a Pediatric Rheumatologist at the University of Minnesota?** | |
| --- | --- | --- |
|  | | This is his/her first visit |
|  | | 0-6 months |
|  | | 7-11 months |
|  | | 1-2 years |
|  | | 3-5 years |
|  | | 6-10 years |
|  | | Greater than 10 years |

| **2.** | **What is/are your child’s rheumatology-related problem(s)? (Choose all that apply.)** | | | |  |  | |
| --- | --- | --- | --- | --- | --- | --- | --- |
|  | | Diagnosis not yet determined |  | Juvenile dermatomyositis (JDM) | | |  |
|  | | I do not know |  | Periodic fever syndrome | | |  |
|  | | Juvenile idiopathic arthritis or juvenile rheumatoid arthritis (JIA/JRA) |  | Inflammatory bowel disease (Crohn’s disease or ulcerative colitis) | | |  |
|  | | Spondyloarthropathy |  | Psoriasis | | |  |
|  | | Reactive arthritis |  | Inflammatory eye disease (uveitis/iritis) | | |  |
|  | | Systemic lupus erythematosus (SLE) |  | Behcet’s disease | | |  |
|  | | Mixed connective tissue disease (MCTD) |  | Amplified musculoskeletal pain | | |  |
|  | | Sjogren’s disease |  | Chronic recurrent multifocal osteomyelitis (CRMO) | | |  |
|  | | Lyme disease |  | Other; please specify:_______________________________ | | |  |

| **3.** | **Are you or your child involved in any organizations or support groups (in-person or on-line) related to your child’s health concerns?** | |  |  |
| --- | --- | --- | --- | --- |
|  | | Yes; please specify which ones:_____________________________________________  (Examples: Arthritis Foundation, JAFN, Walk to Cure JA, Camp Cambria, Lupus Foundation, Cure JM, Facebook groups, etc.) | |  |
|  | | No | |  |

| **4.** | **Does your child have a primary care provider (pediatrician, family practice doctor, etc.)?** | | |
| --- | --- | --- | --- |
|  | | Not sure   \|  \| **My child’s primary care doctor is a . . .** \| \| \| --- \| --- \| --- \| \|  \| \| Pediatrician or pediatric nurse practitioner/physician assistant \| \|  \| \| Family practice doctor or family practice nurse practitioner/physician assistant \| \|  \| \| Other; please specify:______________________________________________ \|  \|  \| **A primary care clinic should provide comprehensive care where your child is treated in a “whole-person” fashion, and the clinic helps coordinate care with specialty doctors. In your experience, how has your child’s primary care clinic done with meeting this expectation?** \| \| \| --- \| --- \| --- \| \|  \| \| 0 = Very poorly 5 = Adequately 10 = Very well    0 ------------------------------------------------------------------------- 10 \| \| |  |
|  | | Yes |  |
|  | | No |  |

|  |  |
| --- | --- |
|  |  |
|  |  |
|  |  |

| **5.** | **Thinking back to when your child first started having concerns, was it difficult or easy to get a referral to Pediatric Rheumatology?**   \| **Can you please share why getting the initial referral was difficult?** \| \| --- \| \| ________________________________________________________ \| \| ________________________________________________________  ________________________________________________________  ________________________________________________________ \| \|  \| | | |
| --- | --- | --- | --- | --- | --- | --- | --- |
|  | | Unsure or do not remember |  |
|  | | Very difficult |  |
|  | | Somewhat difficult |  |
|  | | Neither difficult nor easy |  |
|  | | Somewhat easy |  |
|  | | Very easy |  |

| **6.** | **Please rate to what degree each of the following has been a barrier or obstacle to your child receiving adequate Pediatric Rheumatology care at any point in time. Place an “X” on each line scale to mark your answer.** | |
| --- | --- | --- |
| 0 = No difficulty  Availability of appointment dates/times | | 10 = Difficult enough to stop you from getting health care for your child  -------------------------------------------------------------------------  10  0 |
| Travel time/distance | | ------------------------------------------------------------------------- |
| Need for lodging/housing | | ------------------------------------------------------------------------- |
| Adequate transportation | | ------------------------------------------------------------------------- |
| Driving in the metro area | | ------------------------------------------------------------------------- |
| Direct costs (medical bills, medications, etc.) | | ------------------------------------------------------------------------- |
| Indirect costs (gas, hotel, food, etc.,  NOT including lost work pay) | | ------------------------------------------------------------------------- |
| Parent/caregiver missing work | | ------------------------------------------------------------------------- |
| Child missing school | | ------------------------------------------------------------------------- |
| Arranging care for other children | | ------------------------------------------------------------------------- |
| Insurance approval for visits | | ------------------------------------------------------------------------- |
| Other:_____________________________ | | ------------------------------------------------------------------------- |

| **7.** | **Which one option BEST describes the USUAL amount of time it takes you to travel from your home to your child’s Pediatric Rheumatology appointment?** | |
| --- | --- | --- |
|  | | 0 – 1 hour |
|  | | More than 1 hour |
|  | | More than 2 hours |
|  | | More than 3 hours |
|  | | More than 4 hours |
|  | | More than 6 hours |

|  |  | |
| --- | --- | --- |
|  | |  |
|  | |  |
|  | |  |

| 8. | Regarding your child's Pediatric Rheumatology care at the University of Minnesota, how would you rate your level of satisfaction with your OVERALL experience in each of the following areas: | | | | | | |
| --- | --- | --- | --- | --- | --- | --- | --- |
|  | | Very satisfied | Somewhat satisfied | Neither satisfied nor dissatisfied | Somewhat dissatisfied | Very dissatisfied | Not applicable |
| Scheduling appointments | |  |  |  |  |  |  |
| Appropriately answering your questions about your child | |  |  |  |  |  |  |
| Appropriately answering your child’s own questions | |  |  |  |  |  |  |
| Preparing your child for labs and/or procedures | |  |  |  |  |  |  |
| Performing your child’s labs and/or procedures | |  |  |  |  |  |  |
| Our communication with your child’s primary care doctor | |  |  |  |  |  |  |
| Our communication with your child’s other specialty doctors | |  |  |  |  |  |  |

|  | **If you would like, please share why you were dissatisfied.** |
| --- | --- |
|  | __________________________________________________________________________ |
|  | __________________________________________________________________________  __________________________________________________________________________  __________________________________________________________________________ |
|  |  |

| **9.** | **Do you feel that your child’s Pediatric Rheumatologist considers your and/or your child’s needs, goals, and values when caring for your child?** | |
| --- | --- | --- |
|  | | Always |
|  | | Very often |
|  | | Sometimes |
|  | | Rarely |
|  | | Never |
|  | | Not sure |

| \| **10.** \| **Telemedicine (sometimes referred to as telehealth or e-health) is the use of technology to provide virtual health care at a distance. An example of this would be a clinic visit where the patient is in a clinic closer to home and video/audio technology is used to allow a real-time visit with a doctor at a more distant site. Have you or a close family member or friend ever used telemedicine services?** \| \| \| --- \| --- \| --- \| \|  \| \| Yes \| \|  \| \| No \| \|  \| \| I do not know \| |  |
| --- | --- | --- | --- | --- | --- | --- | --- | --- | --- | --- | --- | --- | --- |

| **11.** | **Which ONE of the following statements BEST matches your views on telemedicine?** | | |
| --- | --- | --- | --- |
| Telemedicine visits are . . . | | | \| **Can you please share why you feel this way?** \| \| --- \| \| _____________________________________________ \| \| _____________________________________________  _____________________________________________  _____________________________________________  _____________________________________________ \| \|  \| |
|  | | . . . better than in-person clinic visits. | |
|  | | . . . equal to in-person clinic visits. | |
|  | | . . . worse than in-person clinic visits. | |
|  | | I do not know enough about telemedicine to decide | |

| **12.** | **Which ONE of the following do you find MOST TRUE of your situation?** | | |
| --- | --- | --- | --- |
| Travel to the Pediatric Rheumatology Clinic is . . . | | |  |
|  | | . . . not an inconvenience, and I would prefer in-person visits. | |
|  | | . . . not an inconvenience, but I would prefer telemedicine options. | |
|  | | . . . inconvenient, but I would prefer in-person visits. | |
|  | | . . . inconvenient, so I would prefer telemedicine options. | |

| **13.** | **In the past 7 days, approximately how many days did you do the following: (Circle appropriate number.)** | |
| --- | --- | --- |
| Use the internet (including on mobile devices such as a cell phone) | | 0 1 2 3 4 5 6 7 |
| Use video chat services to communicate with someone (Examples: Skype, FaceTime, Google Hangout, etc.) | | 0 1 2 3 4 5 6 7 |

| **14.** | **Please rate whether you agree/disagree with the following statement:**  ***“If my child’s Pediatric Rheumatologist had appointments available at a clinic closer to my home, I would be interested in this instead of appointments at the University of Minnesota.”*** | | |
| --- | --- | --- | --- |
|  | | Strongly agree   \| Would you still be interested in this if it meant . . . \| Yes \| No \| Maybe \| \| --- \| --- \| --- \| --- \| \| . . . your child might sometimes see a different Pediatric Rheumatologist than he/she is currently seeing? \|  \|  \|  \| \| . . . instead of a doctor, your child would see a nurse practitioner or physician assistant trained in Pediatric Rheumatology? \|  \|  \|  \| |  |
|  | | Somewhat agree |  |
|  | | Neither agree nor disagree |  |
|  | | Somewhat disagree |  |
|  | | Strongly disagree |  |

|  | **What is/are the reason(s) you are not interested in having your child see his/her Pediatric Rheumatologist at a clinic closer to your home? (Choose all that apply.)** | |
| --- | --- | --- |
|  | | We do not travel very far. |
|  | | The travel is far, but we do not mind. |
|  | | Care at the University of Minnesota is better. |
|  | | My child sees other specialty doctors which requires travel to the University of Minnesota. |
|  | | A closer clinic may not have as many scheduling options (available dates/times) to accommodate  our needs. |
|  | | Other; please specify:_________________________________________________________ |

|  |  |
| --- | --- |
|  |  |

| **15.** | **Please rate whether you agree/disagree with the following statement:**  ***“If there were an Adult Rheumatologist closer to our home who was willing to see my child, I would be interested in this instead of appointments at the University of Minnesota.”*** | |
| --- | --- | --- |
|  | | Strongly agree |
|  | | Somewhat agree |
|  | | Neither agree nor disagree |
|  | | Somewhat disagree |
|  | | Strongly disagree |

| **16.** | **Some clinics are able to work together to make care more convenient. One way to do this is for patients to be seen at a more local clinic such as a primary care clinic or an Adult Rheumatology clinic. The Pediatric Rheumatologist then talks with the local doctor by phone to decide how to treat the patient. This is called “shared care.” Please rate whether you agree/disagree with the following statement:**  ***“If shared care were available, I would be interested in this instead of appointments at the University of Minnesota.”*** | |
| --- | --- | --- |
|  | | Strongly agree |
|  | | Somewhat agree |
|  | | Neither agree nor disagree |
|  | | Somewhat disagree |
|  | | Strongly disagree |

Thank you for taking the time to go through all of the survey questions. We have just a few more questions about you and your child so we can better understand if people have different experiences. Remember, you cannot be identified by your answers to any of the questions in this survey. All of our reports will refer to average responses.

| **1.** | **What is your relationship to the child being seen in the Pediatric Rheumatology clinic today?** | |
| --- | --- | --- |
|  | | Mom or step-mom |
|  | | Dad or step-dad |
|  | | Grandma |
|  | | Grandpa |
|  | | Other; please specify:__________________________________________________________ |

| **2.** | **How old is your child to the nearest whole year?** | | | | | | | | | | |
| --- | --- | --- | --- | --- | --- | --- | --- | --- | --- | --- | --- |
|  | | 0-2 years |  | 3-5 years |  | 6-12 years |  | 13-17 years |  | 18 years or older |  |

| **3.** | **What is your child’s race/ethnicity? (Choose all that apply.)** | | | |  |  | |
| --- | --- | --- | --- | --- | --- | --- | --- |
|  | | White/Caucasian |  | American Indian | | |  |
|  | | Black/African American |  | Asian | | |  |
|  | | Hispanic or Latino |  | Other; please specify:_______________________________ | | |  |

| **4.** | **What is the zip code where your child lives? _______________________________** |
| --- | --- |

| **5.** | **What is the current insurance coverage for your child?** | | | |  |  | |
| --- | --- | --- | --- | --- | --- | --- | --- |
|  | | No insurance |  | Military | | |  |
|  | | Medicaid/medical assistance (MA) |  | Other; please specify:_______________________________ | | |  |
|  | | Private coverage (Examples: Blue Cross, Health Partners, United Health, Medica, Aetna, etc.) |  |  | | |  |

We recognize that our questions do not cover all the aspects related to your child’s care and want to provide you with the opportunity to give any additional feedback that you might have.  The last three questions are open-ended, allowing you to write what you want to say in your own way. This will help us understand other areas that we may not have considered. If you do not have additional feedback, please leave this section blank.

**What barriers or obstacles do you experience when taking care of your child’s health?** ______________________________________________________________________________________________________________________________________________________________________________________

**___________________________________________________________________________________________**

___________________________________________________________________________________________

___________________________________________________________________________________________

___________________________________________________________________________________________

**Please tell us about your concerns related to your child's Pediatric Rheumatology care that were not addressed in this questionnaire.** ______________________________________________________________________________________________________________________________________________________________________________________

**___________________________________________________________________________________________**

___________________________________________________________________________________________

___________________________________________________________________________________________

___________________________________________________________________________________________

**We also want to know what we are doing well. Please share any comments you might have about what makes our clinic or staff excellent.**

______________________________________________________________________________________________________________________________________________________________________________________

**___________________________________________________________________________________________**

___________________________________________________________________________________________

___________________________________________________________________________________________

___________________________________________________________________________________________
