# Supplementary material for: Telemedicine and other care models in pediatric rheumatology: an exploratory study of parents’ perceptions of barriers to care and care preferences
Source: Pediatr Rheumatol Online J. 2017 Jul 11;15:55. doi: 10.1186/s12969-017-0184-y (PMC5504634; doi:10.1186/s12969-017-0184-y)
Supplement: Supplementary file 2 — Acceptability of alternative care models shows variability according to barrier perceptions. This is supplementary table which shows in more details how different barriers to care were scored among those who were/were not interested in alternative care models. (DOCX 17 kb) [file 12969_2017_184_MOESM2_ESM.docx]

| **Additional file 2**: Acceptability of alternative care models shows variability according to barrier perceptions | | | | | | | | | | | | |
| --- | --- | --- | --- | --- | --- | --- | --- | --- | --- | --- | --- | --- |
|  | **Telemedicine preference** | | | **Interest in adult care**** | | | **Interest in shared care**** | | | **Interest in outreach clinic**** | | |
| **Barrier** | **Yes** | **No** | **P-value** | **Yes** | **No** | **P-value** | **Yes** | **No** | **P-value** | **Yes** | **No** | **P-value** |
| Availability of appointment dates/times | 1.00 | 0.50 | 0.9377 | 0.50 | 0.50 | 0.9366 | 0.50 | 0.50 | 0.4627 | 1.00 | 0.00 | 0.0103 |
| Travel time/distance | 5.00 | 2.00 | **0.0142*** | 5.00 | 1.75 | **0.0054*** | 4.50 | 1.00 | **0.0031*** | 4.25 | 0.00 | **<0.0001*** |
| Need for lodging | 0.00 | 0.00 | 0.3615 | 0.00 | 0.00 | 0.6956 | 0.00 | 0.00 | 0.3831 | 0.00 | 0.00 | 0.0229 |
| Adequate transportation | 0.00 | 0.00 | 0.3567 | 0.00 | 0.00 | 0.0482 | 0.00 | 0.00 | 0.0477 | 0.00 | 0.00 | 0.0200 |
| Driving in the metropolitan | 0.50 | 0.50 | 0.6026 | 1.50 | 0.50 | 0.0267 | 2.00 | 0.00 | 0.0168 | 1.75 | 0.00 | **<0.0001*** |
| Direct costs | 1.25 | 0.50 | 0.3395 | 1.00 | 0.50 | 0.2720 | 2.00 | 0.25 | **0.0060*** | 1.00 | 0.00 | **0.0017*** |
| Indirect costs | 1.00 | 0.50 | 0.5897 | 2.00 | 0.00 | **0.0065*** | 1.25 | 0.00 | **0.0071*** | 1.00 | 0.00 | **0.0030*** |
| Parent/guardian missing work | 2.50 | 2.00 | 0.4862 | 3.00 | 1.75 | 0.0837 | 2.50 | 2.00 | 0.2025 | 2.00 | 0.50 | **0.0119*** |
| Child missing school | 1.75 | 2.50 | 0.5756 | 4.00 | 2.00 | 0.1025 | 3.00 | 2.00 | 0.0896 | 3.00 | 1.00 | **0.0059*** |
| Arranging care for other children | 0.25 | 0.00 | 0.9774 | 0.50 | 0.00 | 0.2001 | 0.50 | 0.00 | 0.1739 | 0.50 | 0.00 | 0.0371 |
| Insurance approval for visits | 0.00 | 0.00 | 0.7570 | 0.25 | 0.00 | 0.3443 | 0.00 | 0.00 | 0.2980 | 0.00 | 0.00 | 0.2227 |
| All yes/no values are the median score with 0 = no difficulty to 10 = difficult enough to stop you from getting health care for your child | | | | | | | | | | | | |
| * Denotes statistically significant p-value | | | | | | | | | | | | |
| ** Respondents with neutral interest in these alternative care models are not represented in this table | | | | | | | | | | | | |

Legend: Listed p-values are for the pairwise median barrier score comparisons between those who were (yes) vs were not (no) interested in alternative care models. Notably, certain barriers to care were significantly greater among those expressing interest in alternative care models. For the telemedicine comparison, significance was set at 0.05. For the other care models, significance was set at 0.0167 due to multiple comparisons.
